# Unraveling the Host Genetic Background Effect on Internal Organ Weight Influenced by Obesity and Diabetes Using Collaborative Cross Mice

**DOI:** 10.3390/ijms24098201

**Published:** 2023-05-03

**Authors:** Aya Ghnaim, Iqbal M. Lone, Nadav Ben Nun, Fuad A. Iraqi

**Affiliations:** Department of Clinical Microbiology and Immunology, Sackler Faculty of Medicine, Tel-Aviv University, Tel-Aviv 69978, Israel

**Keywords:** type 2 diabetes mellitus (T2DM), obesity, high fat diet effect on organs’ weight, Collaborative Cross mice, genetic effect, heritability, machine learning

## Abstract

Type 2 diabetes mellitus (T2DM) is a severe chronic epidemic that results from the body’s improper usage of the hormone insulin. Globally, 700 million people are expected to have received a diabetes diagnosis by 2045, according to the International Diabetes Federation (IDF). Cancer and macro- and microvascular illnesses are only a few immediate and long-term issues it could lead to. T2DM accelerates the effect of organ weights by triggering a hyperinflammatory response in the body’s organs, inhibiting tissue repair and resolving inflammation. Understanding how genetic variation translates into different clinical presentations may highlight the mechanisms through which dietary elements may initiate or accelerate inflammatory disease processes and suggest potential disease-prevention techniques. To address the host genetic background effect on the organ weight by utilizing the newly developed mouse model, the Collaborative Cross mice (CC). The study was conducted on 207 genetically different CC mice from 8 CC lines of both sexes. The experiment started with 8-week-old mice for 12 weeks. During this period, one group maintained a standard chow diet (CHD), while the other group maintained a high-fat diet (HFD). In addition, body weight was recorded bi-weekly, and at the end of the study, a glucose tolerance test, as well as tissue collection (liver, spleen, heart), were conducted. Our study observed a strong effect of HFD on blood glucose clearance among different CC lines. The HFD decreased the blood glucose clearance displayed by the significant Area Under Curve (AUC) values in both populations. In addition, variation in body weight changes among the different CC lines in response to HFD. The female liver weight significantly increased compared to males in the overall population when exposed to HFD. Moreover, males showed higher heritability values than females on the same diet. Regardless of the dietary challenge, the liver weight in the overall male population correlated positively with the final body weight. The liver weight results revealed that three different CC lines perform well under classification models. The regression results also varied among organs. Accordingly, the differences among these lines correspond to the genetic variance, and we suspect that some genetic factors invoke different body responses to HFD. Further investigations, such as quantitative trait loci (QTL) analysis and genomic studies, could find these genetic elements. These findings would prove critical factors for developing personalized medicine, as they could indicate future body responses to numerous situations early, thus preventing the development of complex diseases.

## 1. Introduction

The large chronic epidemic known as type 2 diabetes (T2D) arises when the body fails to use insulin. In 2019, 9.3% of the total population worldwide was expected to have diabetes, according to the International Diabetes Federation (IDF). Its number is projected to increase to 10.1% and 10.9% in 2030 and 2045, respectively [1,2]. Due to its increased prevalence, the World Health Organization (WHO) predicts that by 2030, diabetes will be the sixth leading cause of death worldwide [3]. Diabetes can have macrovascular, microvascular, and cancer disorders as short- and long-term effects [4,5]. In previous studies, it was reported that genetics, environment, and other factors play a crucial role in the development of T2D, as well as a prevalent risk factor for obesity [6].

Obesity is an abnormally high buildup of body fat that may have negative health repercussions. Obesity and associated metabolic disorders are linked to diseases that place a heavy strain on the world’s health. Two-thirds of Americans are now considered obese, indicating a sharp increase in its prevalence [7]. Moreover, excess body fat, especially visceral or abdominal fat, might contribute to metabolic syndrome, a condition marked by triglyceride, dyslipidemia, and dysglycemia [8,9,10,11]. Epidemiological and molecular evidence links obesity and metabolic status with inflammation and increased risk of T2D and other chronic diseases [9,10,11], although the mechanisms underlying the association between obesity and these diseases are not fully understood.

After a careful search, several previous studies have confirmed the association of obesity and diabetes with cardiovascular and liver diseases [12,13]. These previous studies reveal a connection between organ weight, obesity, and diabetes; however, they do not touch upon the genetic element involved. Thus, the following study analyzed the genetic component involved in developing T2D and obesity. It has been observed that a high-fat diet (HFD) plays an important part in the disease risk for obesity and T2D through biological mechanisms, including inflammation. The elevated levels of saturated fatty acids (SFA) correlate with the production of inflammatory cytokines [14]. A large body of evidence exists showing that inflammation drives the development of insulin resistance by pro-inflammatory mediators. Exemplifying the role of these pro-inflammatory factors in the pathogenesis of T2D is a mouse model of diet-induced obesity, where inhibition of Tumor necrosis factor alpha (*TNFα*) prevents the onset of obesity-associated insulin resistance [15,16]. Reciprocally, T2D leads to a hyperinflammatory response to the body organs and impairs the resolution of inflammation and tissue repair, accelerating the effect of organ weights. Understanding how the genetic variation translates into different clinical manifestations will highlight pathways through which dietary composition may initiate or accelerate inflammatory disease processes and indicate mechanisms through which disease can potentially be prevented [17]. T2D is complex, and systems genetics is the major approach to understanding the flow of biological information that underlies complex traits [18]. The advantage of systems genetics is that it allows an analysis of molecular interactions in a context that is the most relevant to the clinical trait, namely, multiple genetic perturbations (as in a natural population) rather than an individual genetic perturbation (as in a transgenic mouse) [19]. Comparative mapping showed that mouse models are able to recapitulate human conditions and that the majority of genes in mice are orthologous in the human genome [20].

Some previous studies reveal a connection between organ weight, obesity, and diabetes; however, they do not provide information about the involvement of genetic elements [21]. Thus, to address the host genetic background effect on organ weight by utilizing the newly developed mouse model, known as the Collaborative Cross mice (CC), and to demonstrate the effect of the genetic background on metabolic disturbances and organ weight, CC mice were used in this research designed especially for complex trait analysis [22,23]. In addition, CC mice are the most powerful genetic reference population (GRP) for studying such complex traits [24,25,26]. It is important to mention that the CC mouse will provide a unique and excellent platform and resource for studying the internal organ complications associated with obesity and T2D development due to a high-fat diet and subsequently identifying the genetic factors underlying these Co and multimorbidity. Finally, we used machine learning (ML) methods on our recorded data to classify the different genetic backgrounds under the assessed traits and finally to provide a tool to predict the outcome based on earlier data. ML is a field of study that uses computational algorithms to turn empirical data into models for the purpose of analysis. It allows for the strengthening of statistical and computational approaches that autogenerate the given data [20]. Additionally, ML is implemented to find the relationship between features and their output, known as labels [27]. ML has vastly contributed to medicine in recent years and provides an avenue for the early detection of diseases such as diabetes.

## 2. Results

The following findings were obtained after we evaluated 207 CC mice of both sexes that were kept for 12 weeks in four different food challenge groups by scarifying and weighing the organs.

### 2.1. Variation of Glucose Clearance between Different CC Lines Affected by Diet and Sex

**Figure 1:**
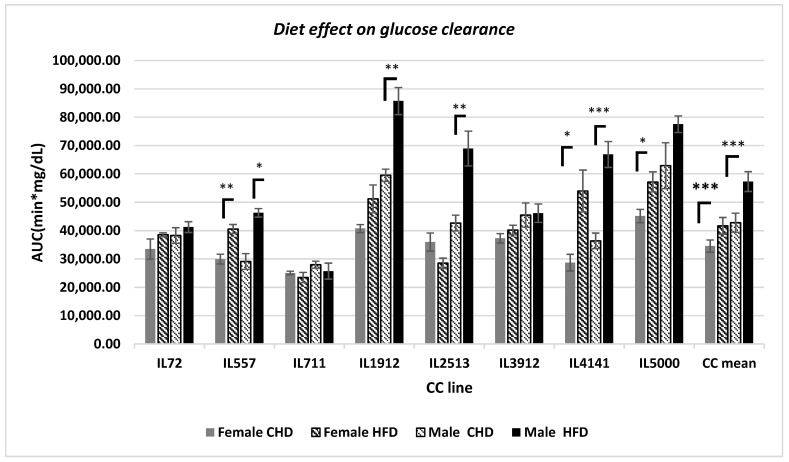
Blood glucose clearance was presented as an area under the curve (min * mg/dL) of 180 min (*y*-axis) glucose tolerance test for eight different CC lines (*x*-axis) of both populations, females and males, divided into four different challenge groups. *, ** and *** indicate significant *p* values, respectively (*p* < 0.05, *p* < 0.01).

In this study, we observed a strong effect of HFD on blood glucose clearance among the different CC lines, as shown in Figure 1. The HFD decreased the blood glucose clearance displayed by the AUC values (significant (*p* < 0.00)) in both populations. Lines IL557 and IL1414 clearly showed a strong effect of HFD on AUC, while in lines IL1912 and IL2513, only males showed significant HFD effects (*p* < 0.05). On the other hand, females of IL 2513 displayed the reverse effect. Three CC lines, IL72, IL711, and IL3912, do not show a large difference between different groups.

### 2.2. Variation in Body Weight Change among Different CC Lines Induced by HFD

**Figure 2:**
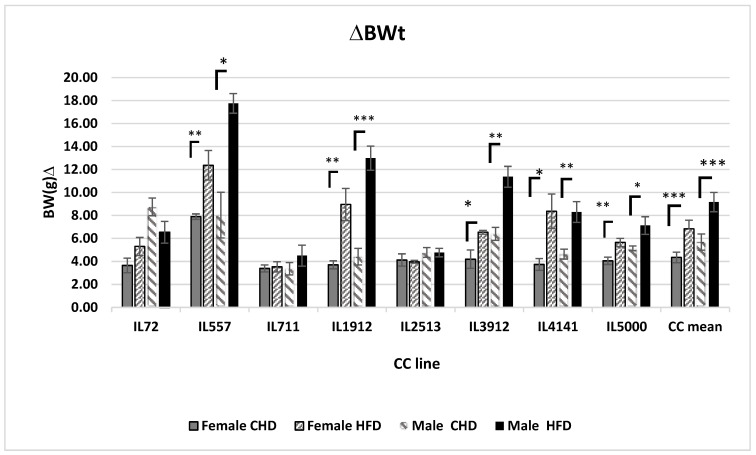
Body weight changes (g) following 12 weeks of diet challenge CHD Vs. HFD for both populations of eight different CC lines (*x*-axis). *y*-axis represents ∆BW(g) calculated as BW12(g)–BW0 (g). *, ** and *** indicate significant *p* values (*p* < 0.05, *p* < 0.01, 0.001), respectively.

The results reveal a variation in body weight changes among the different CC lines as a response to HFD when compared to a CHD group as shown in Figure 2. Five different CC lines: IL557, IL1912, 3912, IL1414 and IL5000 of both sexes showed a significant increase in body weight (*p* < 0.05) as a result of a HFD intake. Overall, HFD significantly (*p* < 0.05) affects weight gain in both populations. However, IL72 responded differently among males of this line, as weight gain was observed as a result of CHD, while among females of the same line, an increase in body weight was recorded on a HFD.

### 2.3. Diet Effect on Organ Weight among Different CC Lines

**Figure 3:**
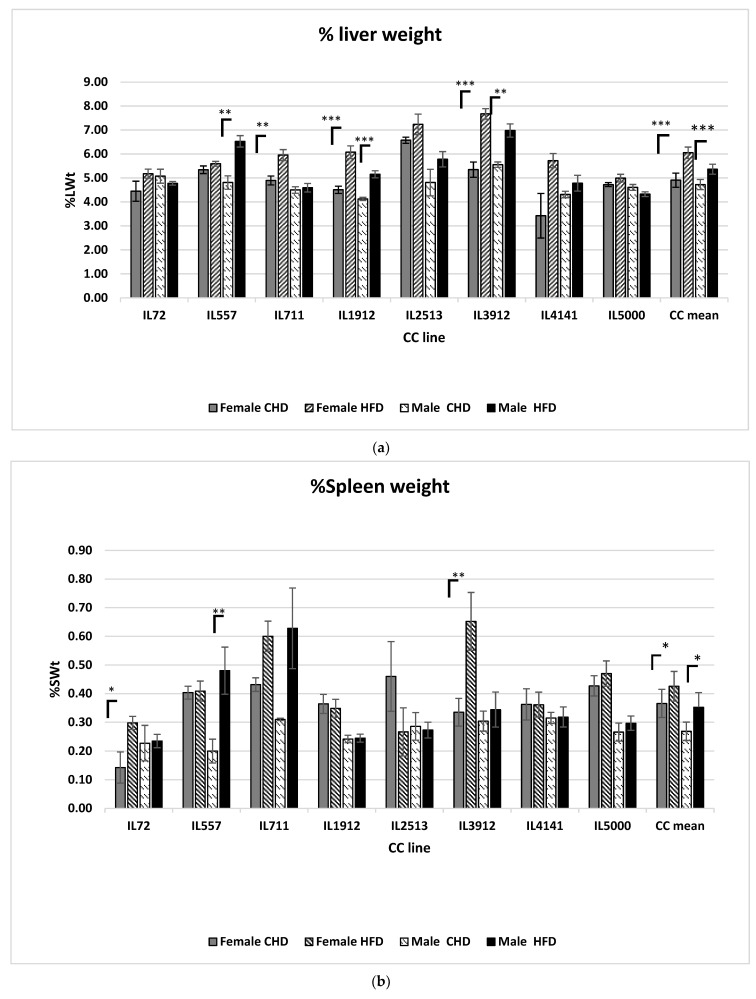

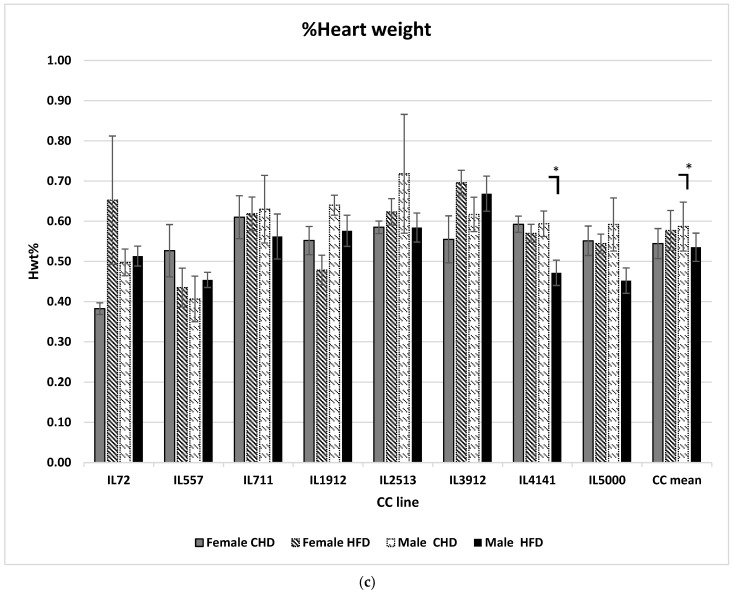
(**a**–**c**) The organ weight in proportion to body weight of eight different CC lines of both sexes maintained on HFD and CHD for 12 weeks. The *x*-axis represents the eight different CC lines, the *y*-axis represents the percent organ weight calculated. (**a**) Represents the liver percentage weight for the four different groups. (**b**) Represents the spleen percentage weight. (**c**) Represents the heart percentage weight for the four different groups. *, ** and *** indicate significant *p* values (*p* < 0.05, *p* < 0.01, 0.001), respectively.

As noted in our results, there is a clear difference in the weight of the different organs between the sexes and in different CC lines as a response to HFD compared to CHD (Figure 3a–c). The female liver weight significantly (*p* < 0.05) increased in comparison to males in overall populations when exposed to HFD (Figure 3a). In both sexes of CC lines IL 3912 and IL 1912, a significant (*p* < 0.05) increase in liver weight was observed as a result of HFD. The line IL 557 responded differently, with only males showing a significant (*p* < 0.05) increase compared to IL 711 where only females responded significantly to HFD and led to an elevated liver weight. The spleen weight increased in both the female and male populations (Figure 3b). Nevertheless, two different female CC lines IL 711 and IL 3912 showed an increase in spleen weight. Moreover, our results showed that the spleen weight in the overall population increased significantly in both sexes in response to HFD. The spleen weight of females in CC lines IL72 and IL3912 showed a significant increase in spleen weight due to HFD, the same result for IL557 in male population was observed. The heart-adjusted weight in the overall male population decreased significantly on HFD compared to CHD group and with the female population on the same diet (Figure 3c). Likewise, the results indicate differences in heart weight among different CC lines in both sexes on the same diet.

### 2.4. Effect of Diet on Organ Weight Changes in Proportion to Body Weight Changes

**Figure 4:**
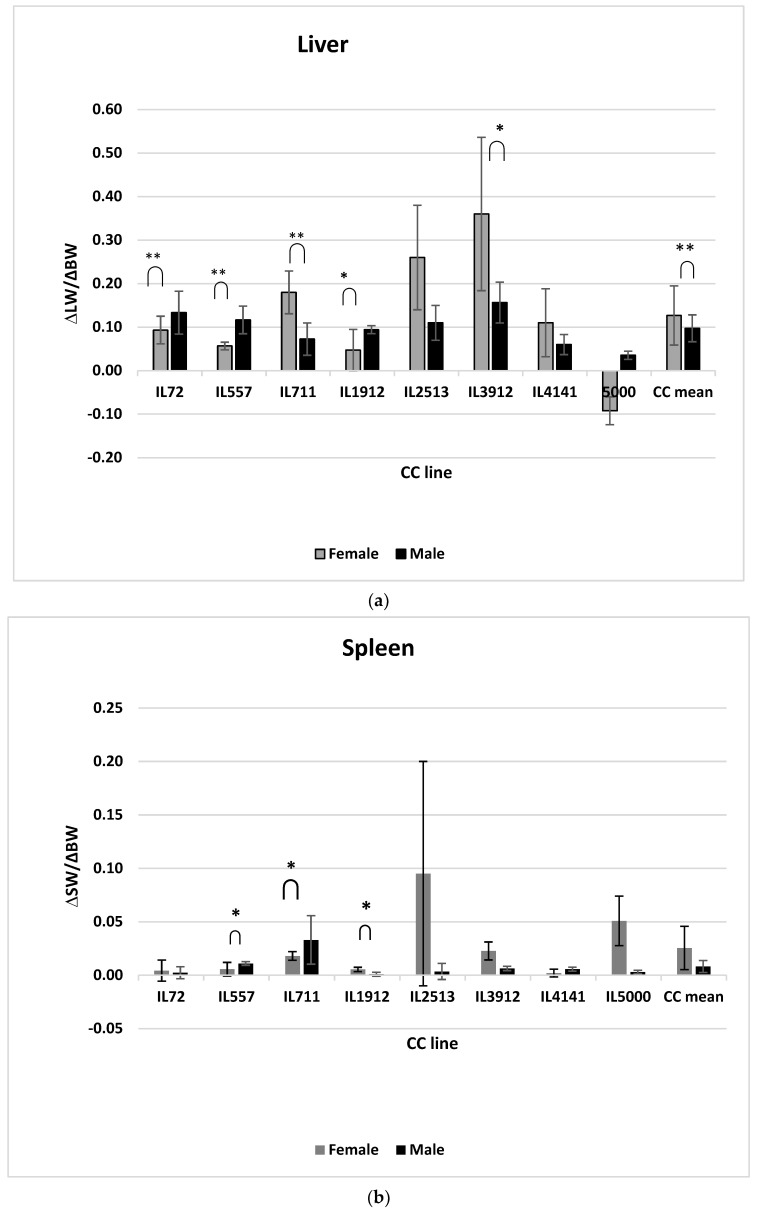

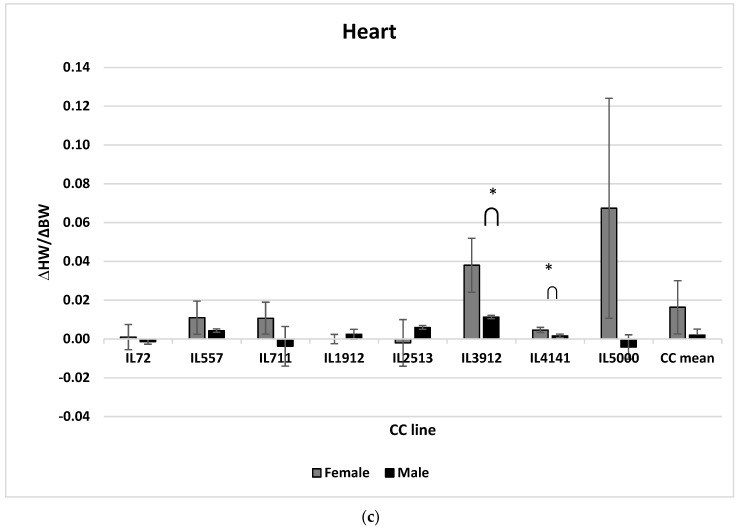
(**a**–**c**) Diet effect on organ weight changes in proportion to body weight changes for eight different CC lines for both sexes. The *x*-axis represents the eight different CC lines for both sexes. The *y*-axis represents delta organ weight divided by delta body weight. (**a**) Represents liver weight changes in proportion to the body weight changes. (**b**) Represents the spleen weight changes in proportion to body weight changes. (**c**) Represents the heart weight changes in proportion to body weight changes. * and ** indicate significant *p* values (*p* < 0.05, *p* < 0.01), respectively.

As presented in Figure 4 the results reflect the diet effect on organ weight changes of both sexes in proportion to body weight changes. The study showed a significant change between male and female liver weight changes (Figure 4a). Furthermore, the results clearly showed the variation among different CC lines in delta liver weight/delta BW in response to diet. The three male CC lines, IL72, IL 557, and IL1912, showed significant change, while the two female CC lines IL711 and IL3912 were also significantly affected by diet. In addition, IL2513 and IL4141 female populations showed a higher ∆ liver weight/∆BW values than male populations. Consequently, the overall female population showed significant (*p* < 0.05) changes in liver weights compared with the overall male population. The spleen results showed the differences among different CC lines and in both sexes (Figure 4b). In male CC lines IL557 and IL711, as well as female line IL1912, ∆spleen weight/∆BW significantly (*p* < 0.05) changed. Regarding the heart, the different CC lines continued to show change in ∆heart weight/∆BW. The female lines IL 3912 and IL1414 significantly (*p* < 0.05) increased more than the male population (Figure 4c).

### 2.5. Heritability and Genetic Coefficient of Variation

The heritability and the genetic coefficient variation were calculated by one-way ANOVA for different tested traits that were calculated separately. Table 1 shows different traits calculated: ∆BW, AUC, actual (liver, spleen, heart) weight, adjusted (% liver, % spleen, % heart) weights for each sex and diet (CHD, HFD), separately; and ∆ LWT/∆BW as well as ∆SWT/∆BW for both sexes, regardless of diet (CHD, HFD). The ∆BW of the female population displayed an estimated value of 0.68 under CHD while 0.54 under HFD, as opposed to the male population ∆BW on HFD value of 0.79 was higher than those maintained on CHD. The AUC values in the male population maintained on HFD were higher at a value of 0.80 when compared to the males on CHD as well as the female population on both diets. Moreover, males showed higher heritability values than females on the same diet. Similarly, the male population displayed higher values of actual spleen weight and adjusted spleen weight on a HFD (0.53, 0.75) than the female population on HFD (0.30, 0.31). The H_2_ estimated values and the CVg values of the effect of diet on liver weight change in proportion to body weight changes in the female population were higher than in male populations: 0.45, 0.33 for H2 and 1.24, 0.41 for CVg, respectively. In contrast, our results displayed low values of the diet effect on spleen changes in proportion to body weight changes in female and male populations (0.19, 0.16), respectively. The CVg values were 0.87 for females and 0.98 for males.

### 2.6. Heatmaps

**Figure 5:**
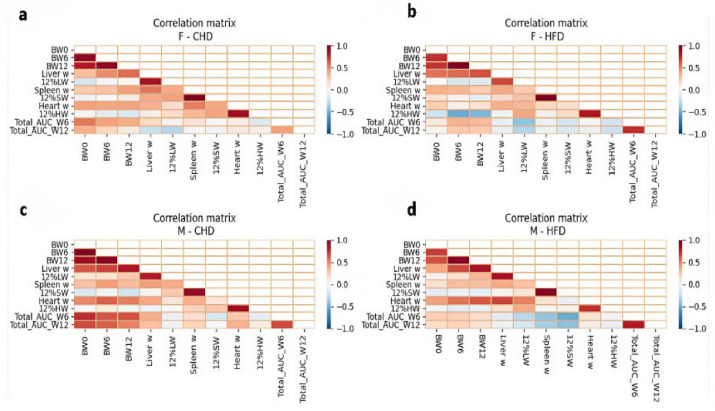
Heat map showing Pearson correlations coefficient for the traits: BW and glucose tolerance, referred to here as AUC, at week 6 and week 12 of the experiment for females (**a**) CHD and (**b**) HFD and males (**c**) CHD and (**d**) HFD, among the different 4 conditions of the experiment. Each map presents both dietary challenges, CHD and HFD, respectively. According to the color key, the correlation coefficient between −1≤ r ≤1 is significant at *p* < 0.05.

From the correlation matrix, we can focus on various relations among the organs and other parameters. Regardless of the dietary challenge, the liver weight in the overall male population correlated positively with the final body weight (0.8) while among the female population it is not highly correlated (Figure 5). The heatmaps of all the individual CC lines have been presented in a Supplementary Figure S1. Additionally, the male population showed a positive correlation between the liver weight and the heart weight as a result of HFD (0.68) compared to a (0.39) on CHD. Furthermore, the different CC lines separately reveal various phenomena among the sex and diet, as well as among the various CC lines. For example, liver and heart weight of different CC lines in the male population maintained on the standard diet (CHD) behave differently, positive correlation shown in IL 557 and IL 1912 (0.89, 0.84), respectively. Positive relation displayed among the female population regardless of the diet challenge in IL711 and IL72 in contrast to line IL 557 which displayed a negative correlation on CHD. Another correlation among some of the CC lines is a relationship between heart weight and spleen weight. The females maintained on CHD display a strong positive correlation in lines IL72, IL557, IL2513, IL5000 (0.91,0.9,0.76,0.78), respectively. Interestingly, CC line IL557 of the male population responded negatively on HFD, while on CHD the response was positive. Furthermore, among lines IL 4141 and IL 72 in the male population the relation between the liver weight and the spleen weight was not affected.

### 2.7. Classification and Regression Models

Our second main aim in this study is to classify and predict the organ weight by using the sex, diet, initial and final body weight, as well as the total AUC values in both week 6 and week 12. In Table 2, adjusted liver weight results revealed that three different CC lines perform well under two classification models with values above 0.8: IL 557, IL 3912, and IL 4141, while IL 72, IL2513, and IL 5000 behave differently and did not perform well under different models with values ranging between 0.16–0.65. Moreover, in Table 2, the results showed that the spleen-adjusted weight of CC line IL 1912 could be classified by three different models with values above 0.8. In addition, the results revealed in Table 2 that the heart-adjusted weight cannot be predicted by any of the used models in all the different CC lines.

### 2.8. Regression–Liver, Spleen, and Heart

The regression results vary among organs and have been presented in Supplementary Table S1a–c for liver weight, spleen weight, and heart weight, respectively. While for adjusted liver weight, we see several lines with mean $R^{2}>0$ (lines IL557, IL711, IL1912, and IL3912), for adjusted heart and spleen weight, our scores are all zeros. In addition, the two models perform differently for each line: for line IL557 we see the top score is achieved by KNN regression (0.26), while for other lines, the linear regression achieved a higher score.

## 3. Discussion

Major worldwide health problems such as obesity and type 2 diabetes (T2D) are associated with metabolic imbalances and may aggravate conditions that result in chronic inflammation. HFD care is the cause of these conditions. Numerous molecular mechanisms have demonstrated that HFD plays a major role in the emergence of obesity and subsequently increases the risk of developing T2D. Additionally, inflammation in the metabolic and adipose tissues is associated with insulin intolerance in both obesity and diabetes [28,29], causing the pancreatic beta cells to start producing more insulin in order to keep blood sugar levels regular, which could result in circulating hyperinsulinemia.

Previous research has identified a number of external risk factors that increase the likelihood that T2D patients will become obese [30]. Results from our laboratory have demonstrated the influence of host genetics on these processes [31]. Additionally, the outcomes from our laboratory showed a range of reactions under the same environmental conditions that were kept on HFD, enabling us to draw conclusions about the influence of the host genetic background [32]. The fact that the sex effect is pervasive in disease development is further evidenced by the different responses that were observed between the sexes [33,34,35].

Earlier reports have shown the correlations among organ weight, body weight, and diabetes; however, the genetic background was not taken into account [36]. This study focused on the role of genetic makeup on organ weight as a result of obesity and diabetes induced by HFD. The variance in genetic makeup and the resulting phenotypes in the study conducted present different responses. Our study has found that the different tested lines responded differently to glucose tolerance tests. These findings are strengthened by previously published work [37]. The diversity in response to glucose tolerance tests stems from the dissimilarity and variation in the genetic background. The glucose clearance in both populations significantly decreased due to HFD intake, while in male population the reduction was more significant; the same result has been demonstrated in previous studies [35,38].

Knowing that the research environment in our study was similar to all the different CC lines and diet groups, the variations obtained should be explained as being due to the effect of the host genetic factor on the organ’s weight, whereas percent liver weight in response to HFD increased significantly in sync with elevation in AUC values and ∆BW in both populations. In CC lines IL557 and IL1912, the results showed that elevation in both parameters did not affect the percent liver weight as seen in CC lines IL5000 and IL1414. Moreover, our results displayed an increase in percent liver weight in response to the elevation of ∆BW and AUC for lines IL3912 and IL2513, respectively. Those two different phenotypes can be classified as early indicators for the severity of liver disease development as non-alcoholic steatohepatitis (NASH), especially those individuals with known susceptible genes for diabetes and obesity as revealed by El-Kader and Ashmawy [39].

Regarding the reduction in the percent heart weight, both of the higher values of AUC and ∆BW affected CC line IL1414 significantly, while other CC lines showed a decrease in this phenotype; therefore, the results obtained were not significant and variable among the different genotypes. For this reason, the chronic inflammation affected by the genetic makeup can affect the heart weight and lead to myocardial damage and, finally, to fibrosis [35,36]. It is important to mention that both sexes behave differently in response to a HFD displayed by organ weight changes and body weight changes. When comparing the female population to the male population, an increase is shown in liver weight in proportion to an increase in body weight change. However, some of the CC lines respond differently to the diet, and the liver weight changes. The influence of genetic makeup is further revealed by the variation in the lines. The male liver increases among lines IL1912, IL72, and IL557 significantly, and among females the same has been observed in lines IL711 and IL3912. Despite the differences in response, the male population showed contraction or shrinkage in the spleen and heart weight as a result of HFD intake, the main reason for BW elevation compared with the female population.

The heatmaps emphasize the difference among the lines. Different values for the same cells across two lines indicate a different body response between the populations. We hypothesized that the difference between the correlation of liver weight and heart weight among lines the IL72, IL557, and IL1912 are a result of the genetic variance, as the overall environmental conditions are identical. The line IL72 has an unexpected pattern of a negative correlation. We must emphasize that it does not necessarily indicate that the growth of a single organ benefits, as significant shrinkage of an organ is mostly undesired, as revealed by Sánchez et al. [40]. We observed that the genetic background of an individual could be useful, or even crucial before assigning a proper treatment as corroborated by Alberts et al. [41]. Similar conclusions can be derived from more organ–organ correlations.

We concluded that the blood glucose levels of these CC lines are affected differently compared to their body weight which has also been observed by Sprague and Arbeláezm [42,43]. For the same lines IL557 and IL711, we observed differences also in the HFD groups. The correlation between the heart’s adjusted weight and final body weight was also observed. For line IL557 the correlation indicates a negative effect and for line IL711 a positive effect was observed. However, since we calculate the adjusted heart weight in response to an increase in body weight, a significant growth in the heart weight resulted in an increase. In other words, a positive correlation indicates an enlargement in heart weight, while a negative value could indicate a minor increase, a steady weight or even a decrease. We thus conclude that for line IL711 obesity could indicate potential heart diseases as also revealed by Powell-Wiley et al. [44], while for line IL557 both factors are probably independent.

The classification results indicate that different lines perform differently under each model. For some lines we get relatively higher AUC values, while for others we get values that are lower than expected. Typically, higher AUC values correspond to better performance of the model. We conclude that the behavior of some lines is probably unpredictable, meaning there are no features that indicate an individual’s adjusted organ weight.

Accordingly, the genetic variance among these lines explains the disparities between them, and it is hypothesized that some genetic characteristics affect how differently various factors react to a high-fat diet [45,46]. These genetic components might be discovered by more research, including quantitative trait loci (QTL) analysis. These findings would serve as important building blocks for the creation of customized treatment since they may be able to predict how the body will react in the future to various circumstances at an early stage, thereby averting the emergence of complex disorders. In addition, the classification was performed based on non-surgical features only. We think that improving these models in the future might prevent some procedures. We anticipate that these discoveries and the pertinent genetic elements will contribute to a reduction in the necessity for surgeries and other checks because mice and humans share a significant portion of their DNA.

## 4. Methods and Material

### 4.1. Ethical Aspects of the Project

It is important to state that all experiments involving animals in this research are compatible with the national standards for care and use of laboratory animals and were approved by the Institutional Animal Care and Use Committee (IACUC) of Tel Aviv University (approval numbers: 01-19-013 and 01-20-015), which adhered to the Israeli guidelines that follow the National Institutes of Health of USA animal care and use protocols.

### 4.2. Study Cohort

This study was conducted on 207 genetically different CC mice originating from 8 CC lines consisting of both sexes. The CC mice were supplied by the animal facility at Tel Aviv University and raised in the facility. They were maintained under the ethical standards of humidity and temperature (21–23 °C). Table 3 represents the method by which the lines were divided into groups (CHD, HFD) and the number of mice of each sex (female and male).

### 4.3. Study Design

The experiment begins when the mice reach the age of 8 weeks and lasts for a duration of 12 weeks. During this period one group of mice was maintained on a standard chow diet (CHD), while the other group was on a high fat diet (HFD). In addition, body weight was reordered bi-weekly, and at the end of the study a glucose tolerance test as well as tissue collection (liver, spleen, heart) was conducted.

### 4.4. Dietary Challenge

Mice were weaned at the age of 4 weeks and maintained until 8 weeks of age on a standard rodent chow diet (TD.2018SC, 18% Kcal from fat, 24% from protein, and 58% from carbohydrates (Teklad Global, Harlan Inc., Madison, WI, USA)). About half of the experimental mice were maintained on CHD throughout the entire duration of the experiment, while the second half was transferred to a Western HFD (TD. 88137, 42.0% Kcal from fat, 15.3% from protein, and 42.7% from carbohydrates (mainly sucrose (Teklad Global, Harlan Inc)) and maintained on it for 12 weeks. The summary table of assessed CC mice in each group represented is in Table 3.

### 4.5. Intraperitoneal Glucose Tolerance Test (IPGTT)

IPGTT was used to detect disturbances in glucose metabolism and reveal instances of T2D among the groups. IPGTT on the experimental and control mice was conducted at four time points during the experiment (weeks 6, 12 starting from week 8 referred to as week 0). The body response to glucose load was assessed by 3 hrs. IPGTT. At each time point, the mice were deprived of food for 6 hrs. (6:00 a.m.–12:00 a.m.), with free access to water. Then, blood glucose (fasting blood glucose) was determined, and a solution of glucose (2.5 gm glucose per kg) was administered by intraperitoneal (IP) injection. Blood glucose levels were measured at different time points during the following 3 hrs. (time 0, 15, 30, 60, 120 and 180 min after glucose injection). After the IPGTT assessment, the mice were returned to their cages with free access to food and water for overnight recovery.

### 4.6. Tissue Collection

At the terminal point of the experiment (20 weeks old mice) the mice from different lines were sacrificed by cervical dislocation, and then tissues including spleen, liver, and heart were extracted and weighed.

### 4.7. Area under the Curve (AUC)

Calculation of AUC was conducted according to the trapezoid role from time 0 to 180 min after glucose injection to quantitatively evaluate glucose clearance activity. AUC between any two-time points is calculated as (time difference in minutes between sequential reads) × (glucose level 1st time point  +  glucose level 2nd time point)/2). The total AUC value of the 180 min IPGTT will be calculated as the sum of the AUC between two-time points, total AUC0-180 = AUC0-15 + AUC15-30 + AUC30-60 + AUC60-120 + AUC120.

### 4.8. Organ Weight in Proportion to Body Weight

The proportion of the organ weight in reference to body weight was calculated

$$\left(\frac{actual\;organ\;weight}{final\;BWt}\;\times \;100\right)$$

### 4.9. Effect of HFD on Change in Organ Weight in Proportion to Changes in Body Weight

The means of the ∆ organ weight was calculated by means of organ weight on HDF subtracted by organ weight mean on CHD, then divided by changes in the mean of ∆ body weight

$$\frac{∆\;organ\;weight\;(HFD-CHD)}{BWt\;(HFD-CHD)}$$

### 4.10. Heritability and Genetic Coefficient of Variation

Heritability measures the fraction of phenotype variability attributed to genetic variation. Here, we used the ANOVA results to calculate the broad-sense heritability using the formula below:

H2=Vg/(Vg+Ve)

where H2 is the heritability, Vg is the genetic variance between the CC lines, and Ve is the environmental variance. Considering the heritability results, we calculated the genetic coefficient of variation (CVg), which indicates the absolute amount of genetic variation. The CVg was calculated using the results of the standard deviation (SD) among the CC lines and trait mean overall CC.

CVg=SD/Mean

For further details on calculation, see [47].

### 4.11. Classification Models

Classification is a data mining (machine learning) method used to predict group connectivity for data instances. Decision tree (DT), random forest (RF), naïve Bayes and k neighbors classifiers were accomplished for further analysis [48,49,50]. They were all applied using the k-fold cross-validation (K = 4) implementation of the Python package scikit-learn. The detailed models are in Lone et al. [18]

### 4.12. Model Validation

Cross-validation is a statistical method of evaluating and comparing learning algorithms by dividing data into two sections: one used to learn or train a model and the other used to validate the model. In typical cross-validation, the training and validation sets must cross over in successive rounds such that each data point has a chance of being validated. The basic form of cross-validation is k-fold cross-validation. Other forms of cross-validation are special cases of k-fold cross-validation or involve repeated rounds of k-fold cross-validation. In k-fold cross-validation, the data is first partitioned into k equally (or nearly equally) sized segments or folds. Subsequently, k iterations of training and validation are performed such that within each iteration, a different data fold is held out for validation, while the remaining k−1 folds are used for learning.

### 4.13. Regression Models

We have used two regression models: linear regression and k-nearest neighbors regression. Each model was run for 100 iterations, and for each iteration, the results were evaluated using $R^{2}$, and a train–test split of 70–30%. The final score we present is the average of those individual scores.

## 5. Conclusions

Accordingly, the differences among these lines correspond to the genetic variance, and we suspect that some genetic factors invoke different body responses to HFD. Further investigations, such as quantitative trait loci (QTL) analysis and genomic studies, could find these genetic elements. These findings would prove critical factors for developing personalized medicine, as they could indicate future body responses to numerous situations early, thus preventing the development of complex diseases.

## Figures and Tables

**Table 1:** Heritability and genetic coefficient of variation values for all tested traits. Heritability and genetic coefficient of variation values for ∆BW, AUC, actual organ weights, percent organ weight, ∆Organ wt/∆Bwt for both sexes and diets.

	Trait	*H2*		*CVg*	
		CHD	HFD	CHD	HFD
**♀**	**∆BW**	0.684	0.572	0.348	0.402
	**AUC**	0.681	0.543	0.205	0.267
	**act-Lwt**	0.497	0.698	0.170	0.212
	**act-Swt**	0.328	0.306	0.211	0.262
	**act-Hwt**	0.155	0.179	0.091	0.102
	**%LWT**	0.543	0.728	0.152	0.143
	**%SWT**	0.322	0.314	1.511	1.222
	**%HWT**	0.131	0.309	0.658	0.975
**♂**	**∆BW**	0.444	0.792	0.276	0.484
	**AUC**	0.593	0.802	0.268	0.347
	**act-Lwt**	0.735	0.835	0.224	0.323
	**act-Swt**	0.092	0.539	0.099	0.386
	**act-Hwt**	0.271	0.629	0.155	0.235
	**%LWT**	0.507	0.750	0.151	0.162
	**%SWT**	0.062	0.750	0.900	2.577
	**%HWT**	0.091	0.363	0.499	1.123
♀	**∆LW/∆BW**	0.45		1.24	
	**∆SW/∆BW**	0.19		0.87	
**♂**	**∆LW/∆BW**	0.33		0.41	
	**∆SW/∆BW**	0.16		0.98	

**Table 2:** Classification results. The input features: sex, diet, initial organ weight, and AUC. The output features: classification of percent organ weight (liver; spleen; and heart)—larger/smaller than the 70th percentile of the data. By using the sex, diet, initial organ weight, and AUC to classify whether percent organ weight would be in the top 30% of the data.

**Percent liver weight**								
Model/Line	IL72	IL557	IL711	IL1912	IL2513	IL3912	IL4141	IL5000
N	18	22	23	34	19	31	25	35
Decision Trees	0.395	0.505	0.531	0.653	0.404	0.442	0.518	0.423
Naïve Bayes	0.25	0.663	0.336	0.631	0.227	0.415	0.431	0.379
K-nearest Neighbors	0.357	0.569	0.597	0.54	0.436	0.339	0.426	0.506
Random Forest	0.362	0.415	0.513	0.687	0.274	0.45	0.465	0.348
**Percent spleen weight**								
Model/Line	IL72	IL557	IL711	IL1912	IL2513	IL3912	IL4141	IL5000
N	18	22	23	34	19	31	25	35
Decision Trees	0.457	0.538	0.499	0.62	0.509	0.488	0.42	0.545
Naïve Bayes	0.387	0.625	0.796	0.848	0.688	0.607	0.349	0.684
K-nearest Neighbors	0.543	0.248	0.331	0.804	0.58	0.563	0.386	0.633
Random Forest	0.443	0.563	0.71	0.81	0.666	0.504	0.365	0.593
**Percent heart weight**								
Model/Line	IL72	IL557	IL711	IL1912	IL2513	IL3912	IL4141	IL5000
N	18	22	23	34	19	31	25	35
Decision Trees	0.361	0.868	0.63	0.779	0.56	0.789	0.847	0.452
Naïve Bayes	0.191	0.946	0.709	0.888	0.335	0.82	0.842	0.566
K-nearest Neighbors	0.326	0.659	0.644	0.471	0.38	0.822	0.478	0.288
Random Forest	0.162	0.921	0.758	0.899	0.493	0.816	0.864	0.377

**Table 3:** The total number of CC mice assessed from eight different CC lines; the number of CC mice used in each diet group (CHD, HFD) of both sexes.

	CHD (11% Fat)		HFD (42% Fat)		
**CC line**	**♀**	**♂**	♀	**♂**	**Total**
**IL72**	4	4	4	6	**18**
**IL557**	6	3	8	5	**22**
**IL711**	7	4	7	5	**23**
**IL1912**	10	9	7	8	**34**
**IL2513**	4	5	3	7	**19**
**IL3912**	4	10	10	7	**31**
**IL4141**	4	8	7	6	**25**
**IL5000**	7	5	14	9	**35**
**Total**	**46**	**48**	**60**	**53**	**207**

## Data Availability

All data presented in this figure is freely available.

## Supplementary Materials

The following supporting information can be downloaded at: https://www.mdpi.com/article/10.3390/ijms24098201/s1.

## Abbreviations

T2DM	Type 2 diabetes mellitus
IDF	International Diabetes Federation
CC	Collaborative Cross mice
CHD	Chow diet
HFD	High-fat diet
T2D	Type 2 diabetes
WHO	World Health Organization
SFA	Saturated fatty acids
GRP	Genetic reference population
ML	Machine learning
IACUC	Institutional Animal Care and Use Committee
IPGTT	Intraperitoneal glucose tolerance test
IP	Intraperitoneal
AUC	Area under the curve
CVg	Genetic coefficient of variation
SD	Standard deviation
DT	Decision tree
RF	Random forest
KNN	k-nearest neighbor
BN	Bayesian network
NASH	Non-alcoholic steatohepatitis
QTL	Quantitative trait loci
