# Supplementary material for: Unraveling the Host Genetic Background Effect on Internal Organ Weight Influenced by Obesity and Diabetes Using Collaborative Cross Mice
Source: Int J Mol Sci. 2023 May 3;24(9):8201. doi: 10.3390/ijms24098201 (PMC10179483; doi:10.3390/ijms24098201)
Supplement: Supplementary file 1 [file ijms-24-08201-s001.zip › ijms-2275865-supplementary.pdf]

The heatmaps of the individual CC lines are presented in this supplementary file.

<u>Heatmaps – per line</u>

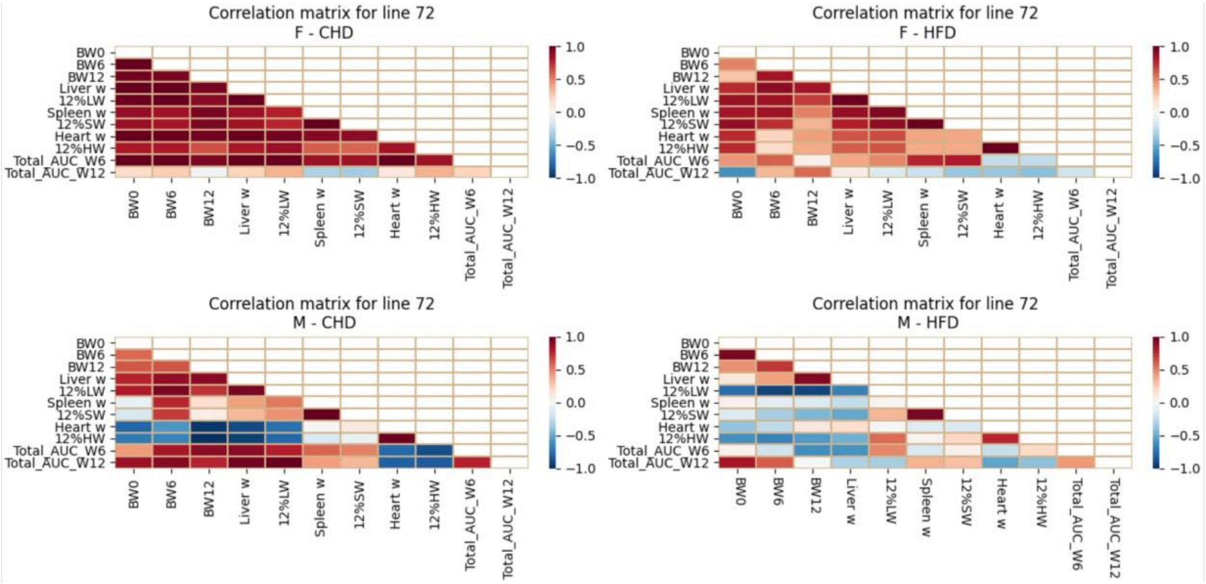

Figure S1 (i): Heat map of Line 72; showing correlations between Sex, Diet and BW and glucose tolerance referred to here as AUC, liver wt, spleen wt, and heart wt at week 6 and week 12 of the experiment for females and males among the different 4 conditions of the experiment. Each map presents a different dietary challenge, CHD and HFD, respectively. According to the color key, the correlation coefficient between $-1 \leq r \leq 1$ is significant at $p<0.05$.

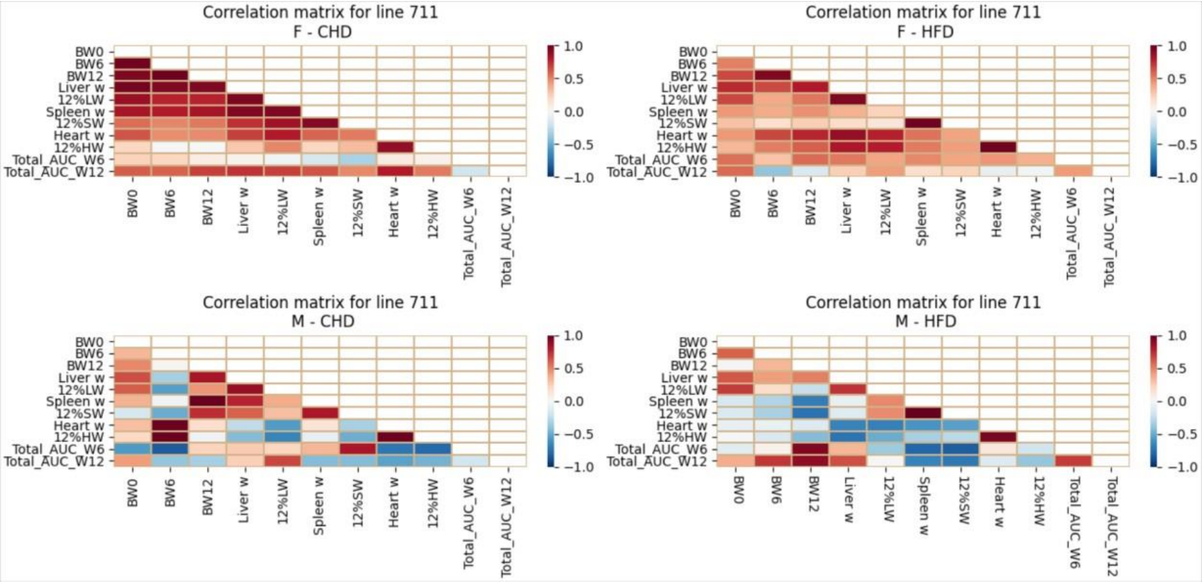

Figure S1 (ii): Heat map of Line 711; showing correlations between Sex, Diet and BW and glucose tolerance referred to here as AUC, liver wt, spleen wt and heart wt at week 6 and week 12 of the experiment for females and males among the different 4 conditions of the experiment. Eachmap presents a different dietary challenge, CHD and HFD, respectively. According to the color key,the correlation coefficient between $-1 \leq r \leq 1$ is significant at $p<0.05$.

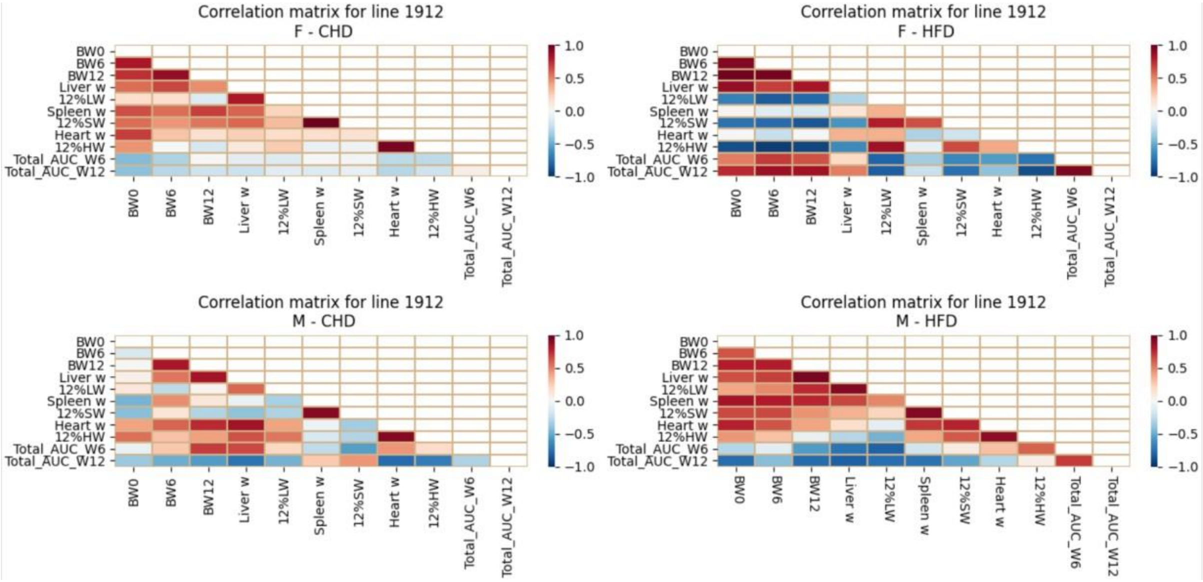

Figure S1 (iii): Heat map of Line 1912; showing correlations between Sex, Diet and BW and glucose tolerance referred to here as AUC, liver wt, spleen wt, and heart wt at week 6 and week 12 of the experiment for females and males among the different 4 conditions of the experiment. Eachmap presents a different dietary challenges, CHD and HFD, respectively. According to the color key,the correlation coefficient between $-1 \leq r \leq 1$ is significant at $p<0.05$.

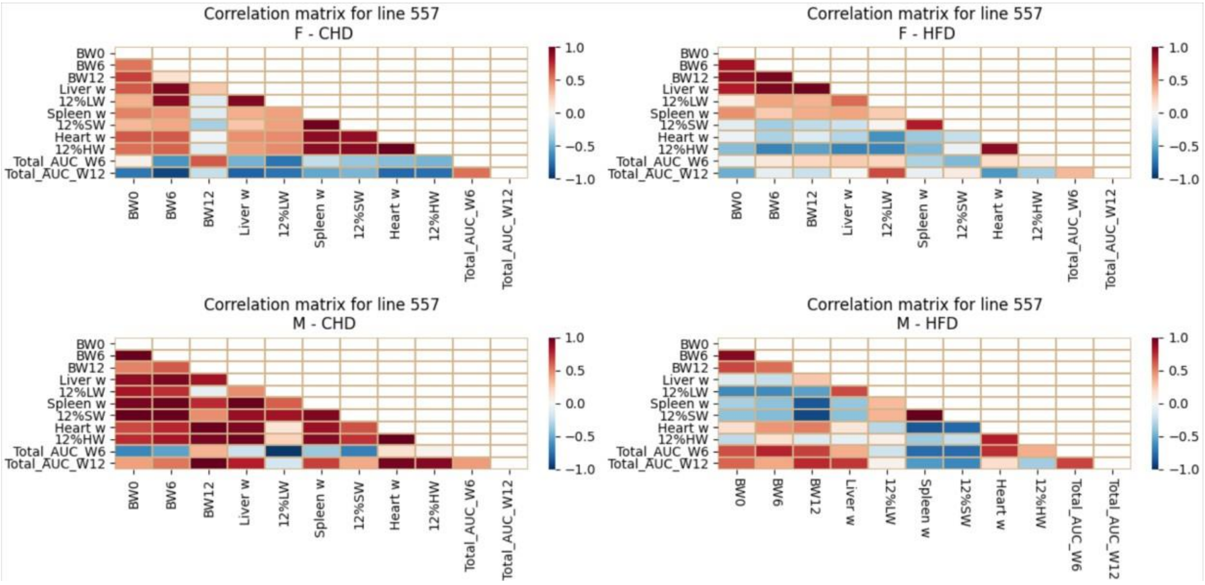

Figure S1 (iv): Heat map of Line 557; showing correlations between Sex, Diet and BW and glucose tolerance referred to here as AUC, liver wt, spleen wt, and heart wt at week 6 and week 12 of the experiment for females and males among the different 4 conditions of the experiment. Eachmap presents a different dietary challenge, CHD and HFD, respectively. According to the color key,the correlation coefficient between -1≤ r ≤1 is significant at p<0.05.

Supplementary Table S1.

Supplementary Table S1a. Percent Liver weight

Line	IL72	IL557	IL711	IL1912	IL2513	IL3912	IL4141	IL5000
N	18	22	23	34	19	31	25	35
KNN regression score	0	0.26	0.09	0.525	0	0.27	0	0
Linear regression score	0	0	0.45	0.58	0	0.37	0	0

Supplementary Table S1a. Classifying the Percent organ weight of the Liver (70th percentile as well) has produced very high values

Supplementary Table S1.

Supplementary Table S1b. Percent Spleen weight

Line	IL72	IL557	IL711	IL1912	IL2513	IL3912	IL4141	IL5000
N	18	22	23	34	19	31	25	35
KNN regression score	0	**0**	0	**0**	0	0	0	0
Linear regression score	0	**0**	**0**	**0**	0	**0**	0	0

Supplementary Table S1b. Classifying the Percent organ weight of Spleen (70th percentile as well) has produced very high values.

Supplementary Table S1.

Supplementary Table S1c. Percent Heart weight

Line	IL72	IL557	IL711	IL1912	IL2513	IL3912	IL4141	IL5000
N	18	22	23	34	19	31	25	35
KNN regression score	0	0	0	0	0	0	0	0
Linear regression score	0	0	0	0.07	0	0	0	0

Supplementary Table S1c. Classifying the Percent organ weight of Heart (70th percentile as well) has produced very high values.
